# Effect of Differences in Access to Screening, Healthcare, and Treatment on Cancer Disparities

**DOI:** 10.3390/ijerph192214747

**Published:** 2022-11-09

**Authors:** Rachel E. Ellsworth

**Affiliations:** 1 Murtha Cancer Center Research Program, Department of Surgery, Uniformed Services University of the Health Sciences, 8901 Rockville Pike, Bethesda, MD 20889, USA; rachel.ellsworth.ctr@usuhs.edu; 2 The Henry M. Jackson Foundation for the Advancement of Military Medicine, Inc., Rockville Pike, Bethesda, MD 20889, USA

Cancer is a heterogeneous disease with over 100 recognized types that differ by organ site and cellular origins. Cancer incidence and survival vary within different populations depending on national origin and geography, race/ethnicity, gender identify, age, income, education, disability, and sexual orientation (www.cancer.gov/about-cancer/understanding/disparities, accessed on 19 October 2022). For example, in 2020, Denmark had the highest overall cancer incidence (https://www.wcrf.org/cancer-trends/global-cancer-data-by-country/, accessed on 19 October 2022). Prostate cancer may be more aggressive in transgender women compared to cisgender men [1], and cancer survival rates are significantly higher in patients with higher incomes, even within universal healthcare systems [2].

Cancer disparities may be attributable to biologic factors, such as the higher rate of mutations in the BRCA1 and BRCA2 genes in people of Ashkenazi Jewish heritage (https://www.cancer.gov/types/breast/hp/breast-ovarian-genetics-pdq, accessed on 19 October 2022) or exposures, such as the higher rate of hepatocellular carcinoma (HCC) associated with hepatitis B virus infection in regions of Africa and East Asia compared to Europe and the United States [3]. Access to medical care, including screening, treatment, and survivorship care, may also lead to disparate incidence, tumor pathology, and outcome in different populations. For example, while rates of mammographic screening are largely similar between Black and White women in the USA, the quality of the screening process, including lower access to digital mammography and dedicated breast imaging specialists, may lead to later-stage diagnosis in minority women [4]. Patients living in high-poverty neighborhoods were significantly more likely to experience treatment delays for HCC than those living in low-poverty neighborhoods [5]. Finally, providers of hospice care demonstrate a lack of awareness regarding the different needs of LGBTQ patients, which may negatively affect their end-of-life experience [6].

An improved understanding of the factors associated with disparate access to cancer care, including socioeconomic status, comorbid conditions, institutional racism, and patient compliance, is critical to reducing cancer burdens and improving outcomes. For this Special Issue, we invite researchers in public health, epidemiology, health economics, biostatistics, psychology, and sociology to submit high-quality empirical papers or systematic reviews that will further broaden our understanding of the healthcare factors that contribute to cancer disparities in different populations.

## Data Availability

Not applicable.
